# Robotic pancreaticoduodenectomy in a case of duodenal gastrointestinal stromal tumor

**DOI:** 10.1186/1477-7819-12-372

**Published:** 2014-12-04

**Authors:** Amilcare Parisi, Jacopo Desiderio, Stefano Trastulli, Veronica Grassi, Francesco Ricci, Federico Farinacci, Alban Cacurri, Elisa Castellani, Alessia Corsi, Claudio Renzi, Francesco Barberini, Vito D’Andrea, Alberto Santoro, Roberto Cirocchi

**Affiliations:** Department of Digestive and Liver Surgery Unit, St. Maria Hospital, Via Tristano di Joannuccio 1, Terni, 05100 Italy; Mininvasive and Robotic Surgery, St. Maria Hospital, University of Perugia, Via Tristano di Joannuccio 1, Terni, 05100 Italy; Department of General and Oncologic Surgery, University of Perugia, Piazzale Gambuli 1, Perugia, 06157 Italy; Department of General Surgery, Sapienza University of Rome, Viale del Policlinico 155, Roma, 00161 Italy

**Keywords:** pancreaticoduodenectomy, robotic technology, GIST

## Abstract

**Background:**

Laparoscopic pancreaticoduodenectomy is rarely performed, and it has not been particularly successful due to its technical complexity. The objective of this study is to highlight how robotic surgery could improve a minimally invasive approach and to expose the usefulness of robotic surgery even in complex surgical procedures.

**Case presentation:**

The surgical technique employed in our center to perform a pancreaticoduodenectomy, which was by means of the da Vinci™ robotic system in order to remove a duodenal gastrointestinal stromal tumor, is reported.

**Conclusions:**

Robotic technology has improved significantly over the traditional laparoscopic approach, representing an evolution of minimally invasive techniques, allowing procedures to be safely performed that are still considered to be scarcely feasible or reproducible.

## Background

Pancreaticoduodenectomy (PD) is one of the most complex interventions in abdominal surgery, still performed by experienced surgeons in specialized centers. The laparoscopic approach to distal pancreaticoduodenectomy has spread significantly, while its use in PD is very rare [1, 2]. Since robotics in digestive surgery represents a recent evolution of the laparoscopic approach, its role and application over the traditional minimally invasive surgery is still debated [3]. A robotic pylorus-preserving pancreaticoduodenectomy performed in our center for the treatment of a duodenal gastrointestinal stromal tumor (GIST) is reported.

## Case presentation

### Case report

A 68-year-old woman was admitted to our department with fatigue, anemia and positive fecal occult blood. The endoscopy showed an enlarged and ulcerated ampulla of Vater. The morphological and immunohistochemical findings were compatible with GIST. Computed tomography (CT) staging confirmed the presence of a parietal, stenotizing polypoid lesion without infiltration of the adjacent tissue. A pylorus-preserving pancreaticoduodenectomy (Traverso-Longmire procedure) using the da Vinci Si™ HD Surgical System robot was proposed.

### Technical notes

**Figure 1:**
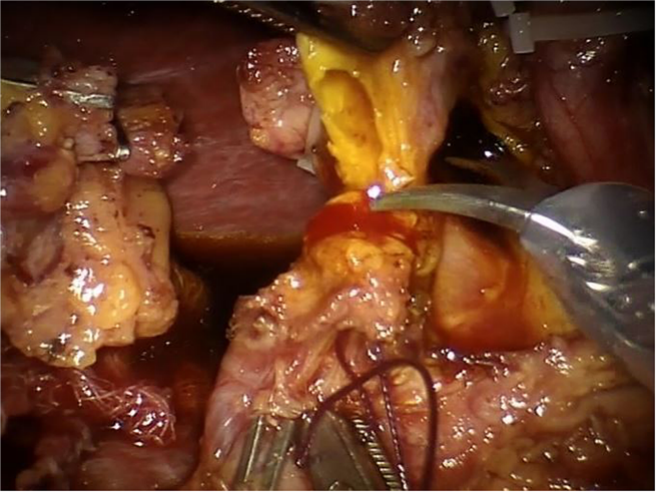
**The bile duct is divided with robotic scissors.**

**Figure 2:**
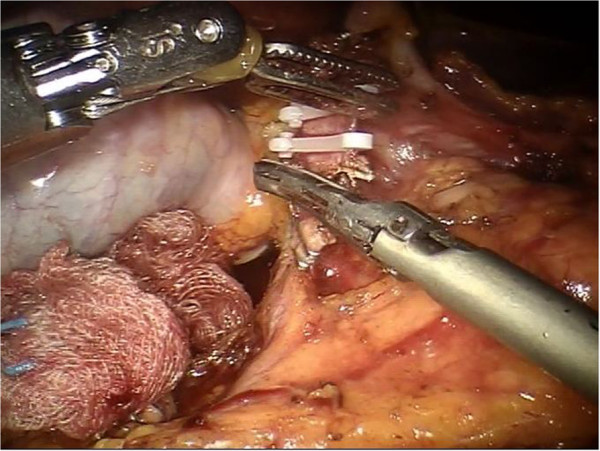
**After the gastroduodenal artery is identified, it is sectioned between the Hem-o-lok using the robotic Ultracision™.**

**Figure 3:**
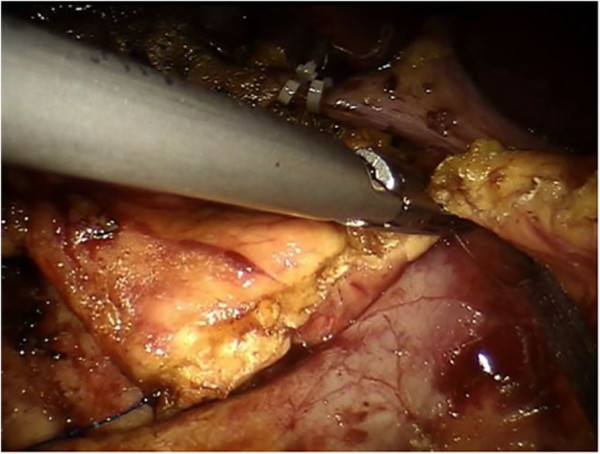
**The dissection of the pancreas is performed with robotic Ultracision™.**

**Figure 4:**
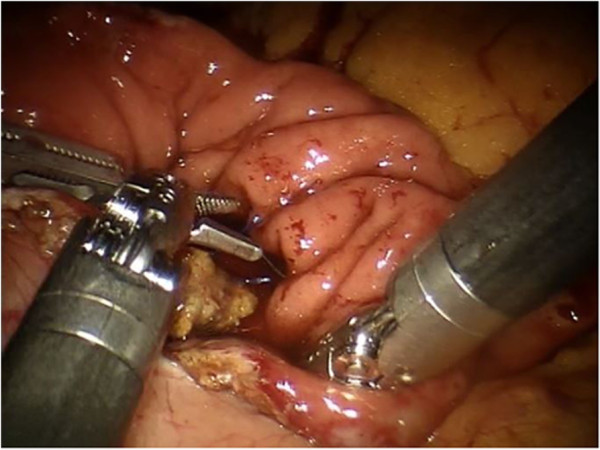
**The pancreaticogastrostomy is performed with a transgastric approach.**

**Figure 5:**
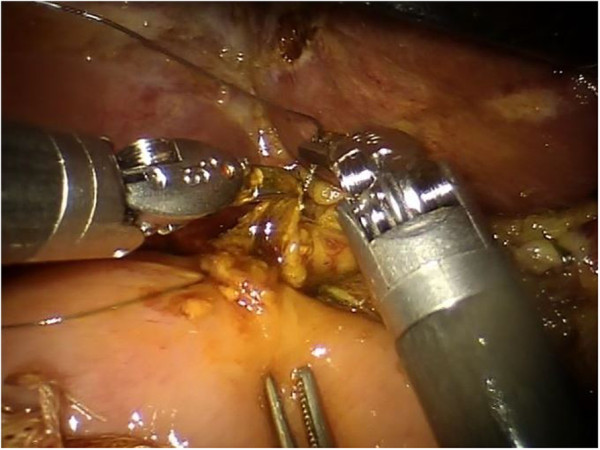
**Execution of the biliary jejunal anastomosis.**

A 20-degree anti-Trendelenburg position is preferred, slightly rotated 10 degrees to the left side. Trocars are placed through the umbilicus following a concave curved line. The optical trocar is inserted about 5 cm to the right of the umbilical line. The first robotic trocar is positioned between the midaxillary and the transverse umbilical line, the second robotic trocar between the right axillary line and the transverse umbilical line, and the third robotic trocar in the right hypochondrium. A 12-mm extra-port is located between the umbilicus and the first robotic trocar. A robotic camera is inserted through the periumbilical trocar port. To access the retrocavity of the epiploon, the gastrocolic ligament is opened outside the gastroepiploic arch. The back wall of the stomach is identified. A Kocher maneuver is performed to release the second and the third portion of the duodenum and views of the inferior vena cava and of the left renal vein. The pancreatic isthmus is identified, a retropancreatic tunnel is created between the posterior face of the pancreas and the superior mesenteric vein (SMV). Then, a retrograde cholecystectomy is performed to allow further mobilization of the duodenal-pancreatic block and to identify the course of the main bile duct, which must be sectioned below the confluence of the cystic duct (Figure 1). The course of the gastroduodenal artery is identified and the artery is sectioned Ultracision™ (Figure 2). The duodenum is sectioned 3 cm below the pylorus with a laparoscopic linear stapler. The retro-pancreatic tunnel is completed, and the pancreas is loaded on a tape. The upper and lower edges of the pancreatic isthmus are closed and a pancreatic dissection is performed with the robotic Ultracision™ (Figure 3). After the course of the SMV and the spleno-mesenteric confluence of the portal trunk are identified, a section of the retroportal pancreatic lamina is performed. A pancreaticogastrostomy is performed via a transgastric approach (Figure 4). The anterior wall of the stomach is opened to allow access to the back wall and the residual pancreas is anastomosed with an interrupted suture technique. Finally, the gastric anterior wall is closed. Then, the ligament of Treiz is identified, and a section of the first jejunal loop is performed. The biliary-jejunal anastomosis is confectioned (Figure 5). A minilaparotomy is performed and a Lap Disc™ is positioned; the termino-lateral duodeno-jejunal anastomosis is closed, and the surgical specimen is retrieved. Two intra-abdominal drains are placed. Surgical time was 510 minutes; blood loss was 250 ml. The postoperative course was fast and smooth. No perioperative complication occurred. On the first post-operative day the Pain Visual Analog Scale (VAS) score was 3 [4]. On the second day fluid intake was restored. Digestive function is recovered on day 3, allowing a solid diet. Bowel functions recovery in day 5 led to the removal of the abdominal drains. The patient was discharged on day 9. The Short Form-12 (SF-12) assessment scale [5] showed a quick return to daily activities. The histopathological examination was group 3 GIST, according to the classification by Miettinen [6]. At 18 months after the operation, there was no recurrence of disease or complications.

## Discussion

Technological advances in recent decades have resulted in significant development of minimally invasive techniques. However, PD still remains a highly demanding procedure and since 1994, when Gagner performed the first PD using a laparoscopic approach, only 146 cases have been reported in the literature [7]. The implementation of robotic systems applied to abdominal surgery has led surgeons keen to use minimally invasive surgery to test a new approach in those procedures. However, to date, robotic PD has not yet been standardized in the literature and the range of surgical techniques employed is wide [8].

Several authors [9, 10] have described a hybrid robotic-laparoscopic-open surgical procedure because robotics was seen initially as complementary to laparoscopy, especially to facilitate the implementation of the visceral anastomosis. Thus, the first procedures to be described in literature consisted in a laparoscopic approach first, with a subsequent robotic reconstructive time. The technique used derives from the one presented by Giulianotti [11], consisting in a fully robotic demolitive and reconstructive time, called robot-assisted PD or simply robotic PD in contrast with the hybrid techniques already mentioned. We believe that the robotic approach is not just complementary, but represents a real evolution compared to the traditional laparoscopic approach, and it is applicable to all stages of PD. According to Giulianotti [3], in robotic surgery the tactile feedback is replaced by visual feedback and the dissection of SMV from pancreatic parenchyma is performed safely and with great precision. The da Vinci™ robotic system increases the surgeon’s dexterity, his ability to perform a precise dissection of the tissues and advanced sutures, and it can replace tactile feed-back with a visual feedback. In this way, it is possible to rebuild the gastrointestinal tract with biliary, pancreatic and gastric hand-sewn 3-4/0 PDS anastomosis and to extract the surgical specimen from a service laparotomy just 5 cm long.

## Conclusions

We believe that the development of robotic technology has increased the indications to perform a minimally invasive approach in complex interventions such as PD, which appears more feasible and safer in selected patients and to experienced surgeons within dedicated teams.

## Consent

Written informed consent was obtained from the patient for the publication of this report and any accompanying images.

## Abbreviations

CT: computed tomography
GIST: gastrointestinal stromal tumor
PD: pancreaticoduodenectomy
SMV: superior mesenteric vein
VAS: Visual Analog Scale
SF-12: Short Form-12.
